# Treatment-seeking behaviour for febrile illness in an area of seasonal malaria transmission in rural Ethiopia

**DOI:** 10.1186/1475-2875-6-49

**Published:** 2007-04-26

**Authors:** Wakgari Deressa

**Affiliations:** 1 Department of Community Health, Faculty of Medicine, Addis Ababa University, Addis Ababa, Ethiopia

## Abstract

**Background:**

Very little is known about the management of malaria and treatment-seeking patterns among children and adults in areas of seasonal malaria transmission particularly in east Africa.

**Objectives:**

The aim of this study was to assess treatment-seeking behaviour for reported malaria among all age groups in an area of seasonal transmission.

**Methods:**

A community-based cross-sectional study was carried out among 2,253 households in 12 randomly selected rural *kebeles *in Adami Tulu district in south-central Ethiopia, during October-November 2003, using a pre-tested interviewer-administered structured questionnaire.

**Results:**

Reported malaria was 14% among 12,225 people assessed during the last 14 days. Family/self-diagnosis was most common and the main first responses included visiting village-based community health workers (CHWs) (33%), public health facility (23%) and private clinic (17%). Home treatment was the least reported first response (3%). Only 13% had sought treatment within the first 24 hours of symptom onset. Early treatment-seeking pattern was reported among those who visited CHWs and practiced home treatment, with more delays among public facility users. Treatment-seeking behaviour was similar in all age groups.

**Conclusion:**

A considerable proportion of visits were made to CHWs and private providers, necessitating the importance of strengthening both community-based interventions and peripheral public and private facilities. Finally, the community should be informed and educated about the importance of early diagnosis and prompt treatment with effective antimalarials.

## Background

Malaria remains the major cause of morbidity and mortality particularly in sub-Saharan Africa [1,2]. In areas with low endemicity, malaria is characterized by frequent and often large-scale epidemics associated with high case fatality rates [3,4]. Although children are at the greatest risk in these areas, all age groups of the population are at risk of severe malaria and death due to lack of protective immunity. Early diagnosis and prompt treatment has been a cornerstone of malaria control [5,6]. However, effective case management partly depends on early recognition of the disease and the subsequent treatment-seeking behaviour from proper care providers.

In the past, treatment-seeking behaviours have been extensively studied in areas of high endemicity [7,8]. Studies conducted in these areas have shown that response to malaria mainly depends on access to a health facility, perceived severity of the illness, perceived quality of care, cultural and traditional beliefs, and knowledge of the symptoms [9]. It is also recognized that treatment of malaria usually starts at home and households seek care outside if home treatment was not successful [10-13], resulting in delays of seeking treatment from a proper care provider.

Despite the extensive work on treatment-seeking behaviours for malaria particularly among children under 5 years of age in areas of intense transmission in sub-Saharan Africa, little is known about the management of malaria and treatment-seeking patterns among children and adults in areas of seasonal malaria transmission [7,14-16]. Due to the severe effects of malaria epidemics, however, increased attention has recently been given to malaria perceptions and practices of the community in areas of low but seasonal transmission [4,7,15-17]. This is of particular relevance to improve early diagnosis and prompt treatment of malaria in epidemic prone areas. The present study assessed the treatment-seeking behaviour of reported malaria in all age groups in an area of seasonal transmission in Ethiopia.

## Methods

### Study area and population

This community-based cross-sectional study was conducted during October-November 2003 in the rural areas of Adami Tulu district in south-central Ethiopia. The capital of the district, Zeway, is located at about 160 kms south-east of Addis Ababa. The district generally lies at an altitude between 1,500 and 1,600 m above sea level and covers an area of 1,403 km^2^. Rainfall is inadequate and seasonal, and lasts between the months of May and November with the greatest intensity during June-August. In 2002, a projected population of the district (from 1994 census) was 145,000 people with a density of 104/km^2^. The population is predominantly subsistence farmers belonging to the Oromo ethnic group and Muslim denomination. Most of the households particularly in rural areas are poor and the majority of the houses are circular *tukuls *with thatched conical roofs.

Malaria transmission in the district is seasonal, with the major transmission from September to December; following a heavy summer rainfall [18]. The study was conducted during peak malaria transmission season (October-November). The district is characterized by frequent occurrence of epidemics often from September to December [19,20]. The parasite prevalence study conducted in all age groups in 1994 showed that the prevalence increased from July (5.4%) to peak in September (12.6%), with an average of 6.8% [18]. *Plasmodium falciparum *and *Plasmodium vivax *constituted 66% and 31% of the infections, respectively [18].

Chloroquine and sulfadoxine-pyrimethamine (SP) resistant falciparum malaria is widespread in the district. In the therapeutic study of SP during October-December 2003 in the district, a total treatment failure rate of 53.4% and late parasitological failure rate of 43.1% were reported against *P. falciparum *on the 14-days follow-up, much higher than the national mean treatment failure rate of 36% [21]. Despite the high rate of resistance, SP was the first-line treatment for uncomplicated falciparum malaria at the time of the study [22]. In 2004, however, Ethiopia changed antimalarial drug policy that adopted artemether-lumefantrine (Coartem^®^) as the first-line treatment for uncomplicated falciparum malaria [23]. Most malaria transmission in the study district is due to *Anopheles arabiensis*, and *Anopheles pharoensis *is considered as a secondary vector [18].

The district had two health centers, three health stations, one malaria control laboratory (MCL) center, three health posts, 15 medium and lower private clinics, 34 community-health workers (CHWs) specifically trained for village-based malaria treatment, one missionary clinic, six drug shops and seven rural drug vendors. Most of these health care facilities were located in Zeway and three other small towns. There was no hospital in the district. As is true in many areas, there could also be a number of unregistered drug outlets in the district.

### Sampling and data collection

The district is administratively organized into 62 rural *kebeles *and four towns. *Kebele *is the lowest administrative unit with a population of 1,000 to 3,000. A list of all rural *kebeles*, including the estimated number of households and population, was obtained. Based on geographical homogeneity and history of malaria problem, all *kebeles *in the district were classified into three strata with the assistance of the District Health Office (DHO) malaria control experts. Finally, four *kebeles *were randomly selected per stratum; thus, making a total of 12 *kebeles *for the study. All households in the selected *kebeles *were included in the survey through house-to-house visits. Household was defined as a group of people including husband, wife, children or others living together sharing the same house.

In each household, the head (or the spouse), or a representative was interviewed using a pre-tested interviewer-administered structured questionnaire about treatment-seeking behaviour for self-reported malaria illness two weeks prior to the interview. The respondents were asked to list all members of their households, and each respondent was asked to think about the last 14 days and indicate any one of the household members who encountered malaria episode. Information was specifically obtained on the symptoms, sources, timing and treatment of reported malaria for all age groups. In order to help the respondent recall the event and practices of seeking treatment, other adult members of the household or sick adults were also participated in answering the questions. For children with reported malaria illness, either the head of household or the mother (or caretaker) or both of them were participated in answering the questions. Twelve local interviewers recruited from the selected *kebeles *and trained for five days collected the data using the local language (i.e., *Afan Oromo*). In the study area, malaria is locally called "*busa*". The author and two health workers from the DHO supervised the data collection.

### Data analysis

Data were entered and analysed using Epi Info software version 6.04d (CDC, Atlanta, GA, USA). Frequencies and proportions were used for the descriptive analysis. Differences in proportions were compared for significance using Chi-square (*X*^2^) test and a *P*-value of < 0.05 was considered significant. The health care providers were classified as (1) public health facilities such as health post, health station, health center, MCL and hospital; (2) private clinic that included high or medium level clinics; (3) CHWs rendering malaria treatment at village level; and (4) home treatment practices. The ages of the reported malaria patients were categorized into children (<5 years and 5–14 years) and adults (≥15 years) for comparison.

### Ethical clearance

The study received ethical approval from the Ethical Committee of the Faculty of Medicine at Addis Ababa University (AAU). Verbal informed consent was obtained from all respondents who participated in the study after explaining the purpose and objectives of the study. Any patient with symptoms suggestive of clinical malaria was treated by SP according to the national guidelines at the time of the study [22]. Patients with other illnesses were advised to seek treatment from appropriate care providers.

## Results

Of 2,253 visited households in 12 rural *kebeles*, data were collected from 2,195 (97%) households. Malaria illness was reported for 14% (n = 1748) of individuals among 12,225 people assessed during 14 days prior to the interview, 70% (n = 1222) of which had recovered on the day of interview. The mean (± SD) age of the patients was 19 (± 15.7) years. The majority of the patients were adults ≥20 years (40%), followed by children <10 year-old (36%). There were about 50% of female patients. The majorities of the reported malaria cases aged ten or above were married (57%), illiterate (71.3%), farmers/housewives (61%) and students (32%).

In all age groups of patients, the most commonly reported symptoms were fever, shivering and chills, loss of appetite, headache and high body temperature (Table 1). Reported shivering and chills among <5 children and adults was higher than that reported among children 5–14 (*P *= 0.013). However, vomiting was more reported among <5 children compared to children between 5–14 year-old and adults ≥15 (19%, 14% Vs. 12%; p=0.018).

**Table 1:** Reported symptoms for perceived malaria illnesses in Adami Tulu district, 2003

		Children			
				
Symptoms^a^	All ages (n = 1748)	<5 years (n = 290)	5–14 years (n = 578)	Adults ≥15 years (n = 880)	*P*-value
Fever	1652 (95%)	227 (96%)	545 (94%)	830 (94%)	NS^b^
Shivering and chills	1510 (86%)	252 (87%)	480 (83%)	778 (88%)	0.013^c^
Loss of appetite	614 (35%)	91 (31%)	196 (34%)	327 (37%)	NS
Headache	603 (34%)	107 (37%)	192 (33%)	304 (35%)	NS
High body temperature	500 (29%)	86 (30%)	157 (27%)	257 (29%)	NS
Vomiting	241 (14%)	54 (19%)	81 (14%)	106 (12%)	0.018^c^
Sleeplessness/restlessness	136 (8%)	17 (6%)	46 (8%)	73 (8%)	NS
Joint pains	13 (0.7%)	2 (0.7%)	3 (0.5%)	8 (0.9%)	NS
General weakness	10 (0.6%)	1 (0.3%)	2 (0.3%)	7 (0.8%)	NS

^a ^Multiple responses possible.

^b ^NS: not significant.

^c ^*P*-value < 0.05 = statistically significant.

The two-week prevalence of reported malaria across the three age group categories indicated that adults' prevalence was higher than that reported for children (Table 2). At the time of the survey, about 77% (n = 1348) of those reported with malaria sought any form of treatment from various sources. The most frequently reported first response was visiting CHWs, followed by public health facilities and private clinics. A considerable proportion (16%) of patients took a second or third action to the same episode of illness since the previous treatment(s) did not provide a cure. Private clinics and public health facilities were the main source of antimalarial treatment by the majority of the patients during the second (41%) and third (35%) visits, respectively. Although symptomatic diagnosis for malaria was reported, 23% of the individuals were neither taken to health facilities nor received any form of home treatment, the main reasons being mild illness (41%), financial constraint (37%), distant health facility (18%), shortage of time due to work overload (3.5%), and the perception that antimalarial drugs were expensive (0.5%).

**Table 2:** Period prevalence of reported malaria and provider choice in Adami Tulu district, 2003

		Children			
				
	All ages	<5 years	5–14 years	Adults ≥15 years	*P*-value
No. interviewed about malaria illness	12225	2249 (19%)	4326 (35%)	5650 (46%)	<0.001^d^
No. with malaria illness in last 14 days (%)	1748 (14%)	290 (13%)	578 (13%)	880 (16%)	<0.001^d^
No. with fever on day of survey (%)	526 (4%)	90 (4%)	180 (4%)	256 (5%)	NS^e^
No. of malaria cases seeking treatment (%)	1348 (77%)	228 (79%)	454 (79%)	666 (76%)	NS
No. first visiting public health facility^a ^(%)	403 (23%)	71(24%)	132 (23%)	200 (23%)	NS
No. first visiting private clinic (%)	294 (17%)	51(18%)	85 (15%)	158 (18%)	NS
No. first visiting CHW^b ^(%)	581 (33%)	97(33%)	210 (36%)	274 (31%)	NS
No. with home treatment as a 1^st ^action^c ^(%)	47 (3%)	6 (2%)	19 (3%)	22 (3%)	NS
No. with two or three treatment sources (%)	272 (16%)	37 (13%)	85 (15%)	150 (17%)	NS

^a ^Health post, health station, health center, malaria control laboratory, and hospital.

^b ^Community Health Worker.

^c ^Initial visits to drug shops or traditional healers excluded from analysis due to small number (n = 23).

^d ^*P *< 0.05 = statistically significant.

^e ^NS: not significant.

Table 3 shows the type of diagnosis for the perceived malaria illness two weeks prior to the survey. Family or self-diagnosis was the most common type of diagnosis within the first 24 hours of the onset of illness. Clinical diagnosis by health workers including CHWs and laboratory diagnosis was infrequently mentioned within the 24 hours of illness onset. The vast majority (76%) did not get any type of treatment or intervention within the first 24 hours of the onset of illness. When respondents were asked about the general diagnosis for this particular reported malaria episode, clinical and laboratory diagnoses increased to 49% and 16%, respectively.

**Table 3:** Type of diagnosis for perceived malaria illnesses in Adami Tulu district, 2003

		Children			
				
**Type of diagnosis**	All ages (n = 1748)	<5 years (n = 290)	5–14 years (n = 578)	Adults ≥15 years (n = 880)	*P*-value
**Within 24 hours after onset of illness^a^**					
Self/family	1280 (73%)	214 (74%)	426 (74%)	640 (73%)	NS^c^
Clinical (health workers and CHW^b^)	207 (12%)	22 (8%)	73 (13%)	112 (13%)	0.048^d^
Laboratory test	69 (4%)	13 (5%)	17 (3%)	39 (4%)	NS
Not diagnosed	314 (18%)	56 (19%)	104 (18%)	154 (18%)	NS
**Overall diagnosis after illness onset^a^**					
Self/family	1360 (78%)	220 (76%)	453 (78%)	687 (78%)	NS
Clinical (health workers and CHW^b^)	864 (49%)	141 (49%)	313 (54%)	410 (47%)	0.017^d^
Laboratory test	278 (16%)	51 (18%)	81 (14%)	146 (17%)	NS

^a ^Multiple responses possible.

^b ^Community Health Worker.

^c ^NS: not significant.

^d ^*P *< 0.05 = statistically significant.

Among those who sought any type of treatment, only 13% had sought treatment within the first 24 hours of onset of illness (Table 4). Most waited until the second day of fever onset before seeking treatment. There were no significant differences in the timings of the first treatment by age. As shown in Table 4, seeking treatment within 24 hours of illness onset was more common among those who practiced home treatment, followed by those who sought treatment from CHWs and private clinics. Treatments from public health facilities were sought lately compared to other sources of treatment.

**Table 4:** Time from symptom onset to seeking first treatment in relation to care providers and age groups, Adami Tulu district, 2003

	No. of days between onset of symptom and seeking first treatment			Total
		
	≤1 day, n (%)	2 days, n (%)	≥3 days, n (%)	
**Care provider^a^**				
Public health facility^b^	36 (9)	148 (37)	219 (54)	403
Private clinic	35 (12)	109 (37)	150 (51)	294
CHW^c^	82 (14)	254 (44)	245 (42)	581
Home treatment	17 (36)	17 (36)	13 (28)	47
**Age group^a^**				
<5 years	29 (13)	93 (41)	103 (46)	225
5–14 years	54 ((12)	172 (39)	220 (49)	446
≥15 years	87 (13)	263 (40)	304 (47)	654
**All**	170 (13)	528 (40)	627 (47)	1325

^a ^Initial visits to drug shops or traditional healers excluded from analysis due to small number (n = 23).

^b ^Health post, health station, health center, malaria control laboratory, and hospital.

^c ^Community Health Worker.

## Discussion

This paper has presented evidence on the treatment-seeking behaviour for reported malaria illness in an area of seasonal transmission in rural Ethiopia. A number of malaria-related symptoms particularly fever, shivering, chills, loss of appetite and headache were presented, which taken together may approximate a clinical diagnosis of malaria although using these symptoms, particularly fever, as a proxy for malaria appears to be neither sensitive nor specific when compared to parasitologically confirmed diagnosis [24,25]. In the present study area, laboratory tests were unavailable at public health facilities such as health posts and health stations. At health center and hospital levels, malaria diagnosis through blood smear examination is usually recommended, but there are occasions that these facilities might not provide blood testing services due to patient overload or shortage of supplies.

The present data is based on self-reported treatment-seeking patterns based on perceived malaria. As a result, all cases reported as malaria may not be malaria, or those not reported as malaria may be truly malaria. The study was conducted during a high malaria transmission season in the area, and its sensitivity would be high considering the transmission season. A study conducted in the adjacent district around Butajira demonstrated 75% sensitivity and 60% specificity using a combination of fever, previous attack of malaria, or the absence of cough during high transmission season [26]. Self/family diagnosis was common among all age groups of our study population, and this pattern of diagnosis is expected because more people in a rural setting hardly access health facilities for better clinical or laboratory diagnosis.

Nevertheless, fever serves as a proxy for malaria both at household level for home management of malaria [27] and peripheral health facilities where diagnosis is made presumptively upon the presence or history of fever [6,23]. However, a recent change of first-line treatment of malaria to artemisinin-based combination therapies (ACTs) in many countries of sub-Saharan Africa has highlighted the potential cost implications of malaria over-diagnosis based upon clinical signs and symptoms [28]. Use of a rapid diagnostic test would help in identifying malaria parasites especially in areas with seasonal malaria transmission where presumptive diagnosis of the disease may be inaccurate but the magnitude of asymptomatic carriers is assumed to be low.

Home treatment in this area of seasonal transmission of malaria was much lower than that observed in areas of similar transmission in Kenya [15] and Uganda [16], and also in areas of high transmission [9,11]. Seeking care from the health facility mainly comes after the failure of care at home [29], or malaria patients might directly seek treatment from health care facilities without the initiation of treatment at home [30]. In a study conducted in east Shewa zone in central Ethiopia, 87% of malaria patients diagnosed at MCLs directly sought treatment without taking anti-malarial drugs at home or prior visit to other health facilities [31]. The present study confirms previous observations in Ethiopia that use of health services for malaria is widely practiced despite the low level of home treatment.

Although public health facilities are assumed to have better institutional organization and capacity that include trained health workers and sustainable supply of drugs, about one-third of the reported malaria cases made first visit to CHWs for malaria treatment. This may not be due to the technical quality of CHWs, but due to their ease accessibility, the availability of antimalarials and their responsiveness to the demand of people. In other words, people in rural areas have little access to public health facilities, despite the inefficient services rendered by the public health-care system [32]. The reasons for the high utilization of private clinics included the perception that they provided high quality services and gave much attention compared to the public services, despite their high cost of treatment. Most private health services are concentrated in the urban centers and small towns. Here it is possible to hypothesize that people are willing to travel long distances to seek health care when quality service is demanded, despite high transportation costs. In the present study area, CHWs were found at a closer distance, but public and private health services were found at a distant for the majority of the rural households. The widespread use of CHWs and private clinics indicates the need to strengthen the community-based malaria control interventions and highlight the importance of private sector for malaria treatment.

The present study findings indicate that delays in seeking treatment for reported malaria is more common among the users of public health facilities, followed by the users of private clinics, compared to the users of CHWs and home treatment. Most malaria deaths among young children occur within 2–3 days after onset of symptoms [33], yet in our study only 13% had sought treatment within 24 hours of onset of symptoms, very much lower than a finding observed in an area of low transmission in Uganda [16] and Kenya [15]. The present study finding has strong implications for improving early diagnosis and prompt treatment with effective antimalarial drug.

Initiation of treatment at home for malaria was started earlier than those sought care from other providers, a finding which is in line with the results of studies conducted elsewhere [10-12]. However, under conditions in which the health service coverage is poor and most facilities are not well strengthened due to lack of resources, seeking treatment from these facilities may not guarantee either prescription of an efficacious antimalarial drug or the correct dosage due to lack of appropriate drug or poor awareness of health workers about the drug policy [16].

## Conclusion

This study has indicated that seeking antimalalria treatment from non-public health facilities such as CHWs and private providers as a first resort to malaria constituted more than half of the visits, necessitating the importance of strengthening community-based interventions and peripheral health facilities. Of special finding is the early treating-seeking pattern of those who visited CHWs and practiced home treatment, with delays most common among the users of the public health facility. Although home-based management of malaria is a promising strategy for improving provision of prompt effective treatment [27,34], the present findings indicate very low level of home treatment, requiring the need for further research. The supply of effective antimalarial drugs such as artemether-lumefantrine to the commonly used care providers is the most important step to reduce the impact of malaria among the rural community. People should be informed and educated about the importance of early diagnosis and prompt treatment with effective antimalarials within 24 hours of symptom onset.

## Competing interests

The author(s) declare that they have no competing interests.

## Authors' contributions

WD was the principal investigator of all aspects of the study from conception and design to the final analysis and preparation of the final manuscript. He is also the guarantor of the paper.
